# Impact of Lithium on the Immune System: An Investigation of T-Cell Subpopulations and Cytokine Responses in Rats

**DOI:** 10.1007/s12011-024-04202-8

**Published:** 2024-05-03

**Authors:** Erdal Matur, Sibel Akyol, Selmin Toplan, Semra Ozdemir, Ibrahim Akyazı, Nuran Darıyerli

**Affiliations:** 1 Department of Physiology, Faculty of Veterinary Medicine, Istanbul University-Cerrahpasa, Istanbul, Turkey; 2 Department of Physiology, Cerrahpasa Medical Faculty, Istanbul University-Cerrahpasa, Istanbul, 34099 Turkey; 3 Department of Biophysics, Cerrahpasa Medical Faculty, Istanbul University-Cerrahpasa, Istanbul, 34099 Turkey

**Keywords:** Lithium, Immune system, Cytokines, Inflammation, Flow cytometry

## Abstract

The aim of this study was to investigate the dose-dependent adverse effects of long-term dietary lithium administration on specific aspects of the defense system in rats. Additionally, the study aimed to explore the inflammatory activities of lithium beyond its recognized anti-inflammatory properties. Forty Wistar Albino rats were involved, which were randomly allocated into the control and four treatment groups. The control group received standard rat feed, and the experimental groups’ diet was added 1 g/kg, 1.4 g/kg, 1.8 g/kg, and 2.2 g/kg lithium bicarbonate, respectively. CD4+, CD8+, and CD161 + cells were assessed by flow cytometry. TNF-α, IFN-γ, IL-1β, and IL-2 and IL-4, IL-6, and IL-10 levels were measured. The proportion of CD4 + cells and the CD4+/CD8 + ratio (*P* = 0.005 and *P* = 0.038, respectively) were reduced with the highest dose of lithium compared to the control group. The data regarding pro-inflammatory cytokines showed a dose-dependent increase in serum TNF-α and IFN-γ levels (*P* = 0.023 and *P* = 0.001, respectively). On the other hand, serum IL-1β and IL-2 levels were decreased in a dose-dependent manner (*P* = 0. 001 and *P* = 0. 001, respectively). As for anti-inflammatory cytokines, a dose-dependent decrease was determined in serum IL-4 level (*P* = 0.002), while no significant changes were noted in IL-6 and IL-10 levels (*P* = 0.507 and *P* = 0.732, respectively). In conclusion, lithium adversely impacted the cellular defense system. Furthermore, apart from its anti-inflammatory properties, lithium exhibited cytokine-mediated inflammatory activities. Therefore, lithium’s potential adverse effects on the immune system should be considered in immunodeficient patients and those with an inflammatory status treated with high doses of lithium.

## Introduction

Lithium salts have long been used as a mood stabilizer in the treatment of psychological disorders, such as bipolar disorder, acute mania, major depression, and schizophrenia in humans [1]. Furthermore, lithium has been recommended for the therapeutic approach to herpes simplex, anorexia, substance abuse, and cluster headaches [2]. Recent studies have demonstrated its antiviral properties [3, 4], and lithium has also been reported to reverse the effect of opioids on vascular endothelium [5].

Since mental disorders treated with lithium require a long-term medication process, its potential adverse effects on organ systems have been a constant concern. Within this context, its potential adverse effects on the liver and kidney functions, as well as its impact on cardiovascular, gastrointestinal, and neuroendocrine systems, have been individually scrutinized [6]. Moreover, it has been reported to affect sexual functions in humans [7]. However, due to the multicomponent complexity of immune reactions, its potential impact on the immune system has yet to be thoroughly investigated. Understanding the pathophysiology of lithium is of critical importance due to the accompanying immune dysregulation detected in patients exposed to long-term lithium therapy [8, 9].

The effects of lithium on the immune system have been extensively studied in previous research. For instance, lithium has been reported to increase the production of granulocyte colony-stimulating factor, activate the bone marrow, and promote the proliferation of stem cells [10]. Additionally, it has been reported to increase the production of neutrophils and macrophages [11]. Furthermore, it has been found to enhance the activity of B cells [12] and stimulate the production of immunoglobulins by B cells [11]. Moreover, studies demonstrated that the effect of lithium is not limited to B cells; it also affects T cells. For instance, Pietruczuk et al. [13] found that lithium inhibits the apoptosis of T cells, while Kibirige et al. [12] implied that it reduces the ratio of suppressive T cells to cytotoxic T cells. Ma et al. [14] demonstrated the inhibition of lactic acid-mediated immune suppression by CD8 + cells, and De Sarno et al. [15] showed that lithium inhibits the activation and proliferation of CD4 + cells. Furthermore, changes in the proportions of CD4+, CD8+, and Th17 T-cell subtypes have been reported in diseases where lithium is used in treatment, such as bipolar disorder [16]. It has also been shown that lithium affects the activity of NK cells depending on whether the application is short or long term [17]. Especially in long-term treatments or in the pathogenesis of chronic diseases, the activity of helper T cells (CD4 + cells), cytotoxic T cells (CD8 + cells), and natural killer cells (CD161 + cells) is important, since these cells play a key role in preventing infectious diseases and regulating inflammatory reactions through the secretion of Th1 and Th2 cytokines [18], particularly during psychological illnesses where the immune system is suppressed or activated [19].

Cytokines are signal molecules regulating local and systemic immune responses [20]. Determining the potential changes in cytokine production is essential in assessing current inflammation status or immune cells’ activities [8]. Cytokine levels varied during the treatment of psychosomatic diseases, including bipolar disorder, where lithium was utilized as a mood stabilizer agent [21]. The levels of some cytokines might even show alterations depending on the episodes of relevant disorders [22]. Previous studies have demonstrated that lithium enhances the production of TNF-α in vitro [23]. Similarly, a dose-dependent increase in TNF-α production has been reported in monocytes obtained from humans treated with lithium [24]. Furthermore, plasma TNF-α levels were found to be significantly elevated in bipolar patients undergoing lithium treatment compared to both medication-free bipolar patients and healthy control subjects [25]. However, despite studies reporting an increase in TNF-α synthesis due to lithium, there are also numerous reports suggesting its reduction [26, 27]. A similar pattern is observed for other cytokines. While some studies indicate that lithium boosts the production of IL-1β, IL-6, and IL-2, there are also numerous studies suggesting a decrease [28].

As a brief review of the literature shows above, the impact of lithium on cellular immune response and cytokine production has been extensively investigated. However, it is evident that the responses of the immune system to lithium, with its numerous components and dynamic nature, can vary depending on the pathophysiological mechanisms of the treated disease. Therefore, further studies are needed to fully understand the effects of lithium on the immune system. For instance, it is worth examining how lithium affects the balance between cells of the specific defense system. Furthermore, the relationship between lithium’s attributed pro-inflammatory and anti-inflammatory properties and cytokines needs to be further explored in depth. A better understanding of the effects of lithium on the immune system will contribute to the expanding literature on the subject for optimizing the dose given in different disease states and providing new treatment options. Therefore, the aim of this study was to investigate the dose-dependent adverse effects of long-term dietary lithium administration on specific aspects of the defense system in rats. Additionally, the study aimed to explore the inflammatory activities of lithium beyond its recognized anti-inflammatory properties.

## Materials and Methods

### Animals and Housing Conditions

The experimental procedures were conducted under the rules of the Local Ethics Committee of Istanbul University (Approval number IU. HADYEK. 2011/131). A total of 40 adult male Wistar Albino rats (weighing 250–300 g) were purchased from the Istanbul University Experimental Medicine Research Institute (DETAE/Istanbul University). The rats were randomly divided into one control and four treatment groups (Group I, Group II, Group III, Group IV), each containing eight animals. Animals were housed in polycarbonate cages with wood chips bedding in a climate-controlled animal room (temperature = 25 ± 2 °C; relative humidity = 50–70% ± 5%), with a 12-h/12-h light/dark cycle. Each cage contained four animals, offering a minimum space of 250 cm^2^ per rat. While the control group was fed with standard commercial rat pellet (DSA, Agrifood Product Inc, Kırıkkale, Turkey), 1 g/kg (Group I), 1.4 g/kg (Group II), 1.8 g/kg (Group III), and 2.2 g/kg (Group IV) lithium bicarbonate (Li_2_CO_3_) (Sigma Chemical Co., St. Louis, MO, USA) were added to the diets of the treatment groups, respectively, for 30 days [29]. The amount of lithium added in feed was adjusted considering the doses used in the literature to reach the lower and upper limits of therapeutic doses in the blood [30, 31]. Lower doses were selected for comparison with therapeutically accepted doses, while higher doses were chosen for the purpose of determining the possible toxic effects of lithium. The rats had ad libitum access to feed and tap water throughout the study.

### Blood Collection and Serum Preparation

At the end of the study, all animals were anesthetized (xylazine/ketamine, 10/75 mg/kg, Bayer, Turkey), and 1 mL of blood was harvested from each rat via cardiac puncture into the EDTA tubes (BD Biosciences, San Jose, CA). Further, 3 mL of blood per animal was collected into anticoagulant-free blood tubes to obtain sera. The anticoagulated blood was immediately transferred to the laboratory for flow cytometric analysis, ensuring the cold chain was maintained. Anticoagulant-free blood samples were centrifuged initially at 3000 × g for 10 min at + 4 °C, and serum samples were obtained. Then, the samples were stored at −80 °C for further analysis.

### Serum Lithium Assay

Serum lithium level was assessed by flame atomic absorption spectrometry (Shimadzu-AA-6800 Spectrophometer, Kyoto, Japan). Before initiating the standard analyses, a standard stock lithium solution (Titrisol 1.000, 0.002 mg/L, Merck, Darmstadt, Germany, Catalog number: 109,934) was prepared and applied for initial calibration. This solution was also utilized as the internal quality standard. The hollow-cathode lamp was used for lithium assay and set at “Background Correction” mode (using a deuterium lamp). Each result was corrected for the appropriate reactive used and the blank matrix. The procedure was run in duplicate to verify the measured lithium concentration, ensuring the difference between the measurements remained within 5%. After each measurement, deionized water was used to rinse the camera and the zero control. Lithium standard stock solution (1.000 mg/L, catalog number 109,934 Merck, Darmstadt, Germany) was applied for the positive control. Assay reliability was tested for every 25 samples [32].

### Flow Cytometric Analysis

#### Monoclonal Antibodies

Mouse anti-rat CD3-FITC (clone 1F4, catalog number: MCA772, Bio Rad), mouse anti-rat CD4-R-PE (clone OX-35, catalog number: MCA153R, Bio Rad), mouse anti-rat CD8-PE (clone OX-8, catalog number: MCA48GA, Bio Rad), and mouse anti-rat CD161 RPE (clone 10/78, catalog number: MCA1427, Bio Rad) antibodies were used for the determination of CD3+, CD4+, CD8+, and CD161 + cells, respectively. Furthermore, appropriate isotopic controls were used to determine the non-specific bindings. All monoclonal antibodies were purchased from Bio-Rad Laboratories, Inc. (CA, USA).

### Cell Staining and Analysis

Monoclonal antibodies were titrated for the determination of the appropriate amount. Red blood cells were lysed before the labeling of the peripheral blood lymphocytes. For this purpose, blood samples of 200 µL were incubated at RT (in 3 mL of freshly prepared 1X FACS Lysing™ solution (Catalog number: 349,202, BD Biosciences, San Jose, CA) for 10 min with occasional shaking. Then, the samples were centrifuged at 300 × g for 5 min. The supernatant was removed, and cell pellets were rinsed twice with 2 mL of PBS solution and resuspended at 1 × 10^6^ cells/mL. A 100 µL of lymphocyte suspension was pipetted into the numbered test tubes, and 20 µL of monoclonal antibodies were added to label CD3+, CD4+, CD8+, and CD161 + cells. After 30 min of incubation at 4 °C in the dark, cells were rinsed with 2 mL of PBS solution by centrifugation at 300 × g. Subsequently, the supernatant was removed, and cell pellets were resuspended with 500 µL of 1/10 diluted CellFIX™ (Catalog number: 340,181, BD Biosciences, San Jose, CA). Flow cytometric analyses were performed using a FACScalibur™ flow cytometry equipped with CellQuest software (BD Biosciences, San Jose, CA). A total of 10,000 events were counted.

### Assessment of Serum Cytokines

Serum GM-CSF, IFN-γ, TNF-α, IL-1β, IL-2, IL-4, IL-6, and IL-10 levels were measured by the xMAP technology (Luminex Corporation, Austin, USA) using the Fluorokine MAP Multiplex Kit (Panel catalog number: LXSARM-08) and corresponding beads set (R&D Systems, Minneapolis, MN, USA). All analyses were performed according to the manufacturer’s instructions.

### Statistical Analysis

Statistical analyses were conducted using the SPSS software package (SPSS for Windows, version 11.5.2.1). The normality of the data was assessed using the Shapiro-Wilk test. For normally distributed data, the ANOVA tests were applied to compare group means, further evaluation of differences among groups was performed by the Tukey HSD test. In cases of non-normally distributed data, the Kruskal-Wallis test was applied for comparisons. Post hoc analyses were carried out for nonparametric data using the Mann-Whitney *U* test. Statistical significance was set at *P* = 0.05.

## Results

Serum lithium levels dose-dependently increased in rats that received a lithium-added diet for 30 days (*P* = 0.001) (Table 1).

**Table 1:** Serum lithium levels in rats given long-term lithium in their diet

Groups	Serum lithium level
Control (0.0 g/kg)	0.002^e^ ± 0.004
Group I (1.0 g/kg)	0.152^d^ ± 0.008
Group II (1.4 g/kg)	0.336^c^ ± 0.016
Group III (1.8 g/kg)	0.458^b^ ± 0.038
Group IV (2.2 g/kg)	1.171^a^ ± 0.122
*P* value	0.001

^a,b,c,d,e^Means with different superscripts within the same column are different, *P* < 0.05

**Fig. 1:**
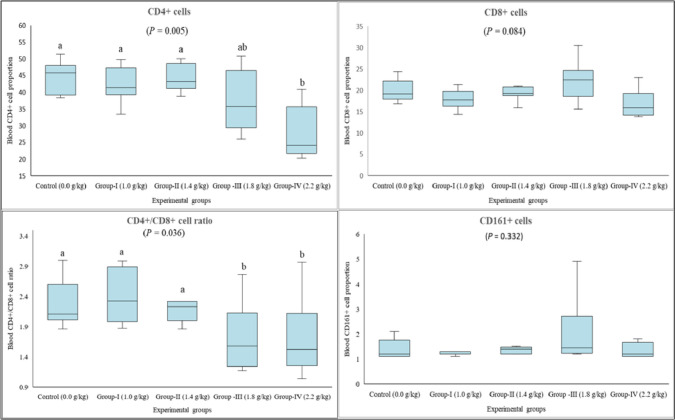
CD4+, CD8+, and CD161 + cell proportion in rats given long-term lithium in their diet. Data are presented by box plots where the central lines represent the median, and the whiskers represent the minimum and maximum values. Different letters above the columns indicate a significant difference between the groups, *P* < 0.05 (*n* = 8)

The proportions of CD4+, CD8+, and CD161 + cells were assessed to determine the potential effect of lithium on immune responses. The data revealed that the proportion of CD4 + cells was reduced with the highest dose of lithium (Group IV) compared to the control group, Group I, and Group II (*P* = 0.005). The proportion of CD8 + cells revealed no significant differences between the control and treatment groups (*P* = 0.084). The CD4+/CD8 + ratio was calculated to determine the potential impact of lithium on the proportional balance among the cells impacting the specific immune system. The findings indicated that the CD4+/CD8 + ratio was significantly lower in Group III and Group IV than in the control and other treatment groups (*P* = 0.038). On the other hand, lithium exerted no statistically significant impact on the CD161 + cells (*P* = 0.332) (Fig. 1).

**Fig. 2:**
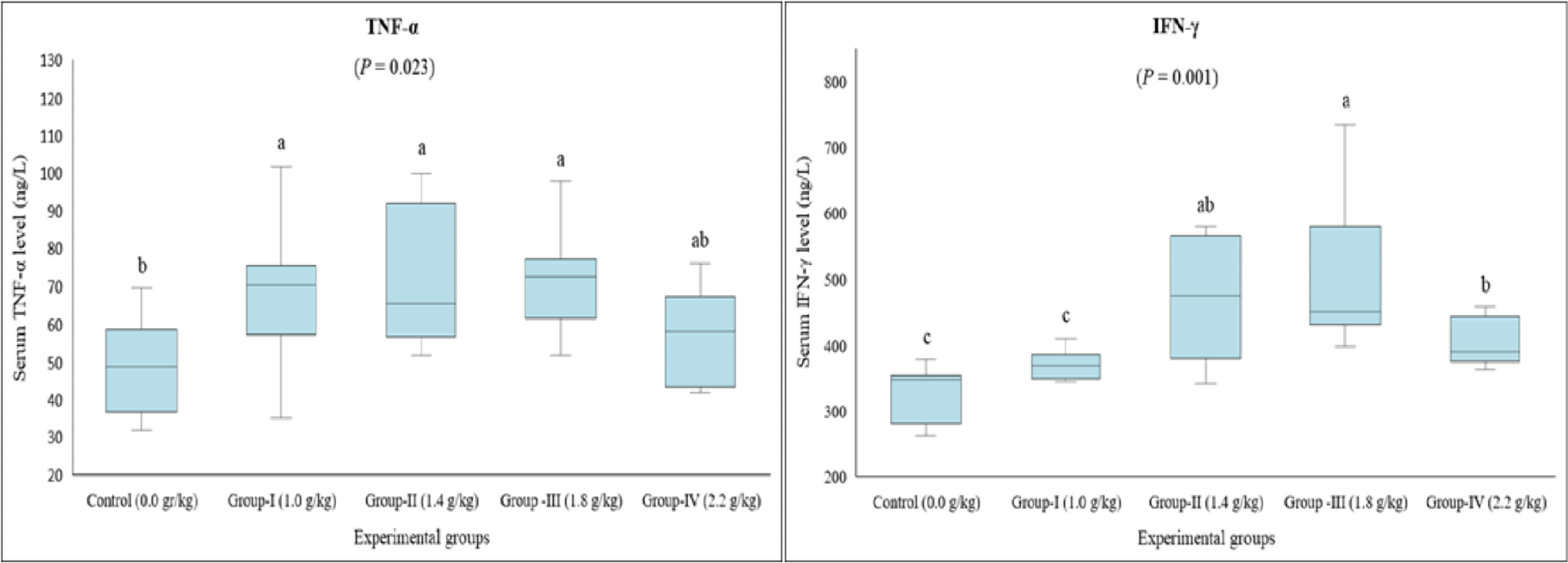
Serum TNF-α and IFN-γ levels in rats given long-term lithium in their diet. Data are presented by box plots where the central lines represent the median, and the whiskers represent the minimum and maximum values. Different letters above the columns indicate a significant difference between the groups, *P* < 0.05 (*n* = 8)

**Fig. 3:**
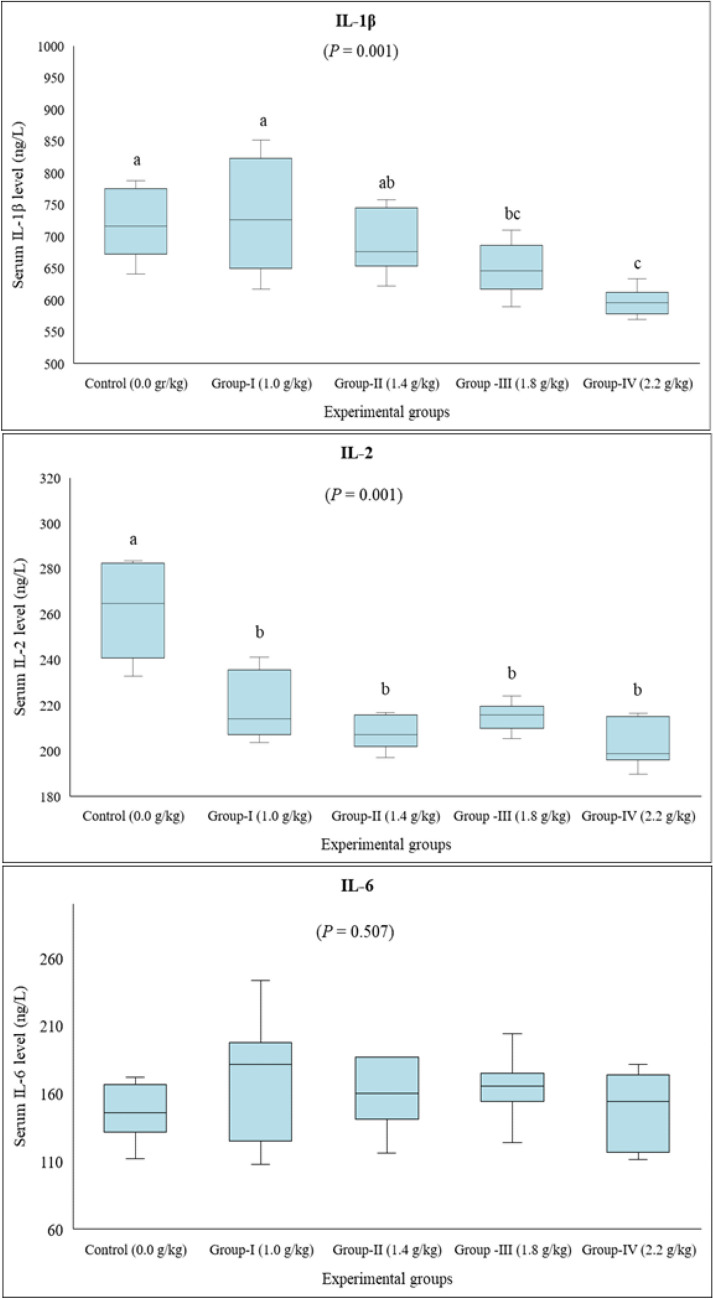
Serum IL-1β, IL-2, and IL-6 levels in rats given long-term lithium in their diet. Data are presented by box plots where the central lines represent the median, and the whiskers represent the minimum and maximum values. Different letters above the columns indicate a significant difference between the groups, *P* < 0.05 (*n* = 8)

TNF-α, IFN-γ, IL-1β, IL-2, and IL-6 levels were measured to determine the potential effect of lithium on pro-inflammatory cytokines. The data revealed that serum TNF-α levels of Group I, Group II, and Group III were higher than that of the control group. As for the highest dose of lithium (Group IV), serum TNF-α level was found to be navigating at a level between those of the control and other treatment groups (*P* = 0.023) (Fig. 2). A similar pattern was observed regarding the serum IFN-γ level, which was increased in all treatment groups except for Group I compared to the control (*P* = 0.001) (Fig. 2). On the contrary, serum IL-1β level decreased dose-dependently. This decrease was statistically significant in Group III and Group IV compared to the control group (*P* = 0.001) (Fig. 3). A similar impact was noted for serum IL-2 levels. All treatment groups revealed lower IL-2 levels than the control group (*P* = 0.001) (Fig. 3). On the other hand, no significant difference was detected between the groups in terms of serum IL-6 (*P* = 0.507) (Fig. 3).

**Fig. 4:**
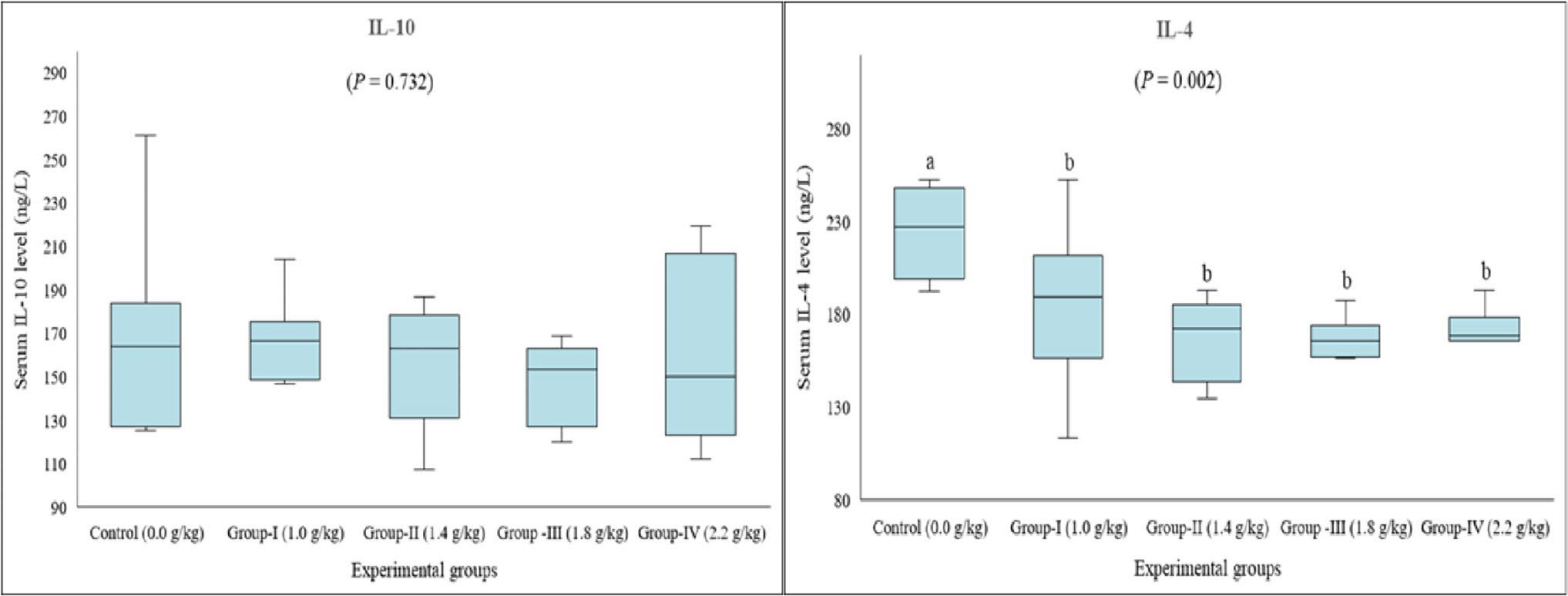
Serum anti-inflammatory cytokine levels in rats given long-term lithium in their diet. Data are presented by box plots where the central lines represent the median, and the whiskers represent the minimum and maximum values. Different letters above the columns indicate a significant difference between the groups, *P* < 0.05 (*n* = 8)

Serum IL-4 and IL-10 levels were measured to assess the potential effect of lithium on anti-inflammatory cytokines. Accordingly, serum IL-4 level was significantly reduced in all treatment groups compared to the control group (*P* = 0.002). No statistically significant differences were noted in serum IL-10 levels between the control and the treatment groups (*P* = 0.732) (Fig. 4).

## Discussion

The main achievement of this study lies in elucidating the dose-dependent adverse effects of long-term dietary lithium administration on specific aspects of the defense system in rats. Furthermore, apart from its anti-inflammatory properties, lithium exhibited cytokine-mediated inflammatory activities. Therefore, we suggest that lithium’s potential adverse effects on the immune system should be considered in immunodeficient patients and those with an inflammatory status treated with high doses of lithium.

The dose-dependent increase in serum lithium levels in the rats receiving a long-term dietary lithium administration revealed accomplished lithium intake. Even though lithium was applied at various concentrations, serum lithium level remained within the therapeutic doses (0.5–1.2 mmol/L) [33], which was significant for monitoring potential adverse effects of clinically indicated doses of lithium.

Lithium salts have been and are currently used as a gold standard to treat various mental disorders, particularly bipolar disorder, to stabilize mood swings and reduce the risk of suicidal attempts [34]. Since these types of disorders usually require long-term medication, its potential adverse impact on the organism is inevitable [35]. The data collected in this study revealed the adverse effects of lithium administered for 1 month to the rats on particular aspects of the defense system in a dose-dependent manner.

Cells recruited in the specific defense system should be present at a specific proportion to achieve an adequate immune response against pathogens. Fagnoni et al. [36] showed an increase in the proportion of CD4 + cells in patients with bipolar disorder treated with lithium, which might suggest the drug’s positive effect on immune responses. However, our data revealed opposing evidence to this finding, where healthy rats administered dietary lithium had a reduced proportion of these cells. In a recent study, lithium was shown to reduce the proliferation of hematopoietic stem cells in mice [37], which was compatible with our findings. Likewise, when the lymphocytes derived from patients with bipolar disorder were treated in vitro with lithium, the cells’ proliferation was reduced, and the cells became more susceptible to apoptosis [13]. Based on the previous data and our findings, we may deduce that lithium exhibits a toxic effect on helper T cells, which is explicit when considering the reduced CD4+/CD8 + ratio (Fig. 1). While an increase in the CD4+/CD8 + ratio indicates a stimulated specific immune system, a reduction, on the contrary, is referred to as an inhibition [38].

The CD161 molecule is mainly expressed on the surface of natural killer cells and is utilized as a marker in flow cytometric analyses to determine peripheral natural killer cells (NKC). These cells play a critical role in recognizing and eliminating neoplastic and virus-infected cells [39]. Patients with bipolar disorder are more likely to develop cancer than the general population [40]; therefore, in the study, the finding that the natural killer cells remained unaffected by lithium administration can be considered a positive outcome.

Inflammatory reactions play a significant role in the pathophysiology of psychological disorders such as bipolar disorder and major depression. Lithium was reported to have attenuated accelerated inflammation in these patients [16]. Lithium’s anti-inflammatory effect is mainly attributed to its inhibition of glycogen synthase kinase-3 beta (GSK-3β) enzyme and associated inflammatory mediators [41]; however, previous studies demonstrated that its anti-inflammatory effect is not only restricted to these mediators but also associated with cytokines. When lithium was administered in vitro to the LPS-induced cells, the secretion of pro-inflammatory cytokines, such as TNF-α and IL-6, was reduced [26, 42], and contrastingly, the production of IL-10, which is an anti-inflammatory cytokine, increased [43], thus suggesting the likely cytokine-mediated anti-inflammatory properties of lithium [44]. On the contrary, in our study, lithium reduced the production of pro-inflammatory cytokines such as IL-1β and IL-2. The levels of these cytokines were reported to be elevated in patients with bipolar disorder, which is involved in the pathogenesis of this disorder [45]. Therefore, in our study, lithium reduced the IL-1β and IL-2 levels in a dose-dependent manner, and this can be considered a positive impact. Yet, unlike the previous studies [26, 42], the production of pro-inflammatory cytokines such as TNF-α and IFN-γ increased, while the anti-inflammatory cytokine IL-4 production fell with the administration of lithium to the non-LPS-induced healthy rats, which might indicate an inflammatory effect of lithium, contradicting the findings of studies suggesting anti-inflammatory properties for lithium.

Another interesting finding in our study was that lithium increased the secretion of some anti-inflammatory cytokines while decreasing others. The decrease in IL-2 and IL-4 levels in lithium-administered rats was associated with the decrease in the proportion of CD4 + cells since IL-2 and IL-4 found in the peripheral blood are mainly secreted by CD4 + cells [46, 47]. We assumed lithium reduced IL-2 and IL4 production by impacting the CD4 + cells. A recent study revealed lithium’s inhibiting pro-caspase-1 [48]. IL-1β is secreted as inactive pro-IL-1β by monocytes and macrophages, and active IL-1β is produced with this molecule’s breakdown by pro-caspase-1 [49]. Therefore, the decrease in IL-1β level was associated with the lithium’s inhibiting pro-caspas-1. On the other hand, unlike in the previous studies [26, 42], the underlying mechanism of the increased TNF-α and IFN-γ production in our study could not be clarified based on our current data. However, previous studies have shown that lithium may modulate TNF-α production through different mechanisms. The best known of these is that it inhibits the GSK-3α enzyme. It is reported that when this enzyme is inhibited, TNF-α production via the p38/MNKI/eIF4E pathway is stimulated at the post-transcriptional stage [50]. It has been reported that the stimulating effect of lithium on TNF-α production may also occur through mitogen-activated protein kinase-extracellular signal-regulating kinase (MEK-ERK), independent of GSK-3 inhibition [51]. In our present study, the increase in the level of IFN-γ may be related to microRNA-29 (miR-29) expression because it had been reported that miR-29 is involved in the regulation of IFN-γ production [52], and lithium is effective in regulating miR-29 expression [53].

Helper and cytotoxic T cells should be present in a sufficient number and proportion to generate adequate immune responses against pathogens. Herein, chronic lithium administration dose-dependently inhibited helper T cells and impaired the balance between helper and cytotoxic T cells, which, in return, adversely impacted the defense system. Furthermore, lithium was observed to exhibit cytokine-mediated inflammatory effects along with its anti-inflammatory properties. Therefore, the potential adverse effects of lithium should be taken into consideration while treating inflammatory disorders, particularly with high doses of the drug. Further studies should be conducted to elucidate lithium’s other potential effects on the immune system.

## Data Availability

No datasets were generated or analyzed during the current study.
